# Do holograms develop hypertension?

**DOI:** 10.1038/s41371-022-00728-3

**Published:** 2022-08-13

**Authors:** Sarah E. F. Nichol

**Affiliations:** Institute of Cardiovascular and Medical Sciences, University of Glasgow, Glasgow, G12 8TA UK

**Keywords:** Hypertension, Disease prevention

*In fair Glasgow, where we lay our scene*,

*A civil difference in blood pressure breaks new mutiny;*

*The maple dominion better than the auld enemy, it seems*,

*Two lands both with shared history*.

*Eight minds here deliberate, and hope to unveil*,

*Reasons for observations this paper does bring;*

*And though doubt in its reflections should prevail*,

*A gamble, a wager, may yet do the right thing*.

*Is now the two thousand words of our page;*

*The which if you with patient eyes attend*,

*What here shall miss, their toil shall strive to mend*.

**Fig. 1:**
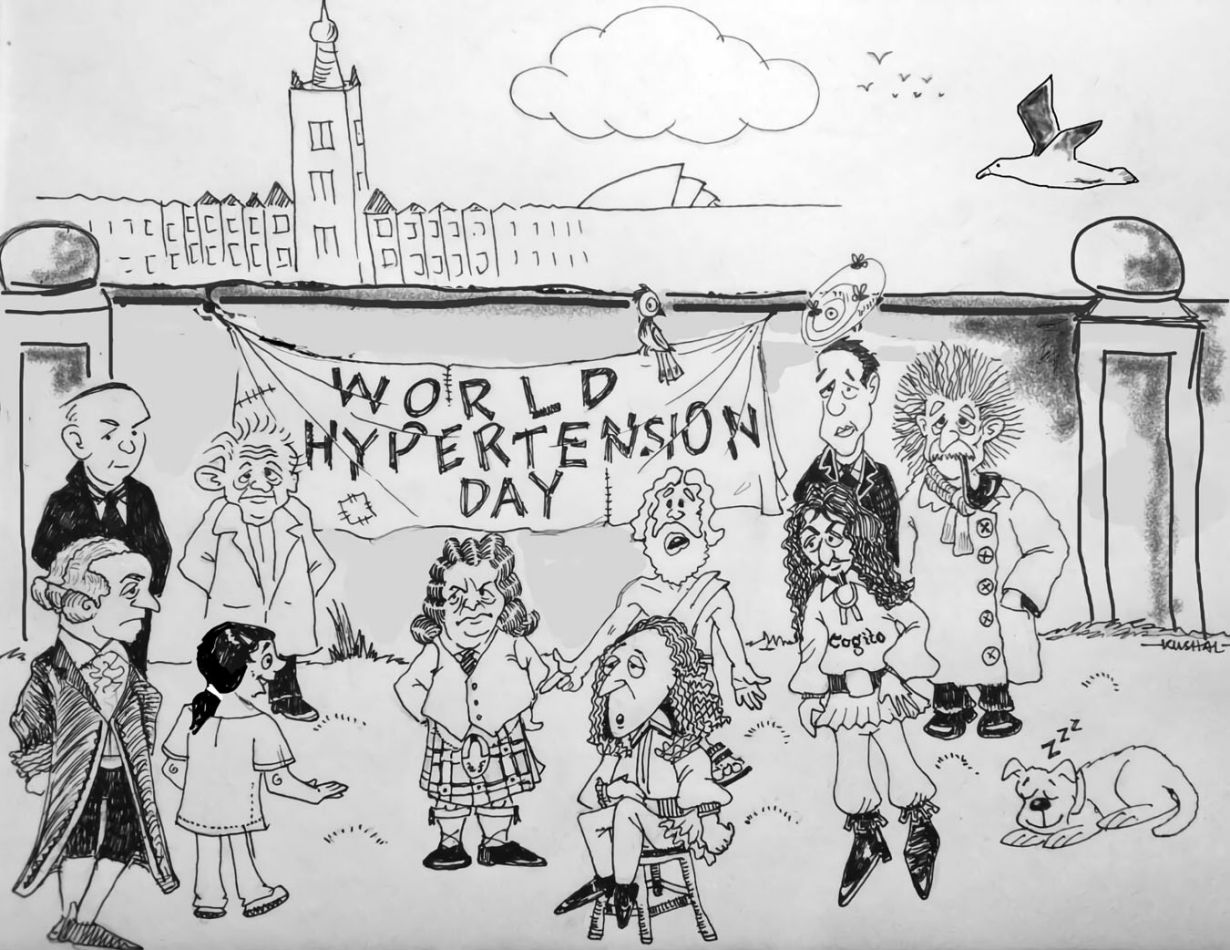
A huddle of experts and philosophers dissecting the BIHS challenge. George Pickering, David Hume, René Descartes, Karl Popper, Immanuel Kant, Plato and Blaise Pascal discuss the essay topic with Sarah Nichol while Einstein and Bohr look on. Illustration by Dr Kushal K Choudhuri.

Sarah: Hey guys, I’ve decided to enter the annual British and Irish Hypertension Society’s (BIHS) essay competition. This year the topic is ‘throughout adult life blood pressure (BP) levels in Canada are substantially lower than in England’, inferred from a paper by Joffres et al. [1]. The challenge is to discuss possible explanations for these observations, and I was wondering if I could discuss some ideas with you? (Fig. 1).

George: Of course, I’ve dabbled in hypertension research [2]. This looks like a cross-sectional analysis of national health survey data from three countries - England, USA and Canada. The results relevant to your question are those indicating that compared to the English, the Canadians have lower BP in all age groups from 20 to 80 years, along with greater hypertension awareness and treatment levels.

David: Och, aye. The truth of this statement is not self-evident [3]. However, the paper provides empirical evidence founded on fact and observation, hence the inductive inference in this statement reflects reality.

René: Sorry to interject, in what sense are the data presented ‘true’? [4]. England and Canada had a respective population of 51 million and 33 million at the time of this study. Therefore, it’s hard for me to believe that a few thousand individuals are truly representative of each population. I’m not an expert like George, but I see that there are also discrepancies in BP measurement in the two countries. How can we be certain of anything if there are problems with the data?

George: Whilst no experimental study is perfect, this sampling is a well-established method to infer information about a population, without having to investigate every individual. Cartesian doubt can only take us so far!

René: J’en ai marre! A mutual admiration society of two empiricists. You measure BP in a small sample, extrapolate it to the whole country and then claim that this is reality. Incroyable!

George: René, we are here to help Sarah, a *tabula rasa* [5], understand the complexities and responsibilities of empirical studies. You do expose an important flaw in the empiricist world view namely, that data is everything and consequently, that data is power. All inference from data is only as good as the quality of the data, and many empiricists are guilty of misinterpretation or overinterpretation of weak data [6]. These weaknesses bring investigators’ beliefs into their interpretation, transforming correlations into generalisations. These are the attendant risks of empiricism, and they have the potential to harm people through direct or indirect exhortations to change behaviour based on flawed science.

Sarah: I’m confused. Are the results of this paper invalid?

René: Oui, and all the inferences from these measurements are also invalid.

David: That is not entirely correct. Inductive inferences based on data always require a modicum of belief [2].

George: Let me try and explain. You are attempting to establish the veracity of the statement that BP levels in Canada are substantially lower than in England throughout adult life. We are presented with a scientific paper with evidentiary data supporting this claim. If we assume that the data in the study are representative of the population, then surely the inference must be true. Quod erat demonstrandum. But is it really proven? René and David are both right in their contentions, and herein lies the challenge that the BIHS has set out for you to solve.

Sarah: Gosh, I understand now. I should not accept the results in this paper at face value, but critically check the validity of the results. The authors do list possible reasons for the observed differences. In 2000, Canada launched the Canadian Hypertension Education Programme (CHEP) to address the shortfalls in national hypertension management. This consisted of annual mailshots to the public as well as free online educational resources promoting dietary salt restriction and other healthy lifestyle choices. Clearly, these actions had an impact on the population, evidenced by increased screening and treatment of hypertension post-rollout of CHEP [7]. I suppose this would support the statement.

David: By Jove, the lassie has got it. Karl, what do you say?

Karl: You cannot prove anything is true by induction. Only by a process of refutation can you establish veracity [8].

George: Ok Sarah, let us try Karl’s refutation method. Can we think of any reason why the difference in BP between England and Canada may not be true? England launched a nationwide salt reduction programme in 2003 to reduce salt in processed foods, primarily aimed to reduce hypertension incidence. Between 2003 and 2011, national salt intake in England decreased by 15 per cent and Health Survey for England over the same period showed national average BP decreased from 129/74 to 126/73 [9]. So, Canada is not alone in implementing public health policies for hypertension and this raises some doubts—but does not imply a full refutation—about the reported differences in BP between the two countries.

Sarah: Also, René brought up the issue of different BP measurement devices used in England and Canada. Are we just comparing apples and oranges?

George: Another good point. The different measurement methods could certainly affect the observations. The Canadian survey used a fully automated device which measures unattended BP, whereas in England BP was measured by a nurse using a semi-automated device. Removing the ‘white coat effect’ could have significantly affected the Canadian BP readings and indicate that the observed difference with England is spurious. Recently, the SPRINT trial was subject to similar criticism that SPRINT’s unattended BP measurement wrongly indicated a greater level of BP lowering [10]. However, a post-hoc study found that the SPRINT triallists used both unattended and attended BP devices, and average follow-up BP levels were comparable across both methods [11]. You can see the Karl’s falsification paradigm in action here.

Sarah: Curiouser and curiouser. Aren’t lifestyle factors such as salt and alcohol intake, smoking, physical inactivity, and stress also implicated in hypertension? [12]. I cannot see any mention of these lifestyle factors being considered in the analysis. Furthermore, I cannot see how one can extrapolate differences in BP measured at one time point to BP across an individual’s lifetime.

René: Exactement, Sarah! With very little effort you have identified the flaws in the data and its interpretation, demolishing the assertion that BP across the life-course is lower in Canadians compared to the English.

Immanuel: Du Heißluftgebläse! René, you are a dogmatic rationalist. Sarah, you must understand in your search for causation, synthetic a priori is the route to substantive knowledge [13].

Sarah: Meh!

David: You are as obscure as ever, Immanuel.

George: You see, Sarah? Scientific research has far-reaching consequences. Scientists have a responsibility to obtain a true result, and also try to establish causal pathways to explain their findings. Hypertension is a minefield. We diagnose hypertension based on BP, but BP is a trait influenced by multiple physiological and environmental interacting pathways [12]. This complicates inductive reasoning about any observed differences in BP. This is where Karl’s idea of falsification is relevant. To differentiate science from pseudoscience, we need to show that any claim is resistant to falsification despite repeated attempts. We cannot accept any assertion as true until it has gone through this process. But falsification cannot address the elephant in the room: is BP the right metric to target interventions given its complex underpinning architecture? This appears to be reductionism taken to the extreme.

Sarah: Do you mean that using BP to diagnose hypertension is simply utilitarian—it is an artificial construct to enable doctors to select individuals for treatment without knowing what the underlying cause is?

George: That is reality. We treat hypertension because there is compelling evidence from randomised controlled trials that reducing BP with drugs will protect from cardiovascular events and improve survival even if the underlying cause is not known [14]. Indeed, the value of treating hypertension is reflected in the National Institute for Health and Clinical Excellence statement that treating hypertension is now cheaper than doing nothing [15].

Sarah: So, what you are saying is there are many inapparent factors contributing to the observations in question. Be that as it may, it does not bode well for an individual to be exposed to a drug that is inappropriate for them. It is overkill to prescribe a drug when simple lifestyle measures may be all that is required for the causal mechanism operative in an individual.

Plato: Hey Sarah, Plato here. You are right to question this level of reductionism in the treatment of hypertension. To me, this is reminiscent of cave-dwellers watching shadows of people outside and creating their own version of reality [16]. Say a cave-dweller leaves the cave and sees the reality of the outside world, and then tries to explain what they have seen to their fellow cave-dwellers: they would be shunned and ignored!

George: Without all the necessary information on confounding variables, we struggle to make conclusions about any differences between the two countries. Furthermore, in these cross-sectional studies, we identify correlations not causations, and inferring a causal link between observed differences and health outcomes is erroneous.

Plato: Sarah, the data that you have from the paper does not represent reality, it is a mere observation of reflections of reality.

(Einstein and Bohr walk by)

Niels: Hey Albert, they are discussing holograms. Can holograms measure their BP?

Albert: Höchst interessant… a Gedankenexperiment measuring BP in a train travelling at the speed of light….

Niels: Shut up Albert.

Sarah: There is so much to consider, and I feel like I’m no closer to explaining these observations to the BIHS!

Blaise: Sarah, do not worry, all is not lost. Let us bet that BP is higher in England than in Canada across the life-course. If we bet on the proposition being true, then the effect will be initiation of effective hypertension screening and management along with positive lifestyle measures. If BP in England is truly higher, then the population benefits. If the proposition is false, there will still be large ancillary benefits such as reduction in the risk of other diseases, such as obesity, diabetes, and cancer, along with better cardiovascular health. Either way brings benefit, perhaps with a little minor additional cost of BP screening and antihypertensive medications. The gamble of believing that BP levels in England are higher is worth a lot more than not believing the results and demanding more studies [17].

Immanuel: I concur. Remember though, this is not just about betting on hypertension management for personal gain—it is about societal responsibility. This is a categorical imperative [18] for affected individuals to do what is right and reduce their BP. It would have significant positive impact on the healthcare system, productivity, and economy, and the burden of hypertension on society means that it is everyone’s moral duty to manage it effectively.

Sarah: Okay, thank you everyone for sharing your thoughts and insights. I now understand the challenges and complexities of conducting valid scientific studies and the power of science on the individual and society. Scientists must aim to produce evidence that maximises benefit and minimises risk, while recognising the difficulties in determining what may be acceptable benefit and risk for an individual or community. As an asymptomatic disease of ever-increasing prevalence, hypertension continues to challenge healthcare providers. Improving antihypertensive prescribing, awareness of positive lifestyle choices and BP control on a national basis would translate into improved clinical outcomes and reduce associated risks to both the patient and society.

All: Good luck Sarah!

**Fig. 2:**
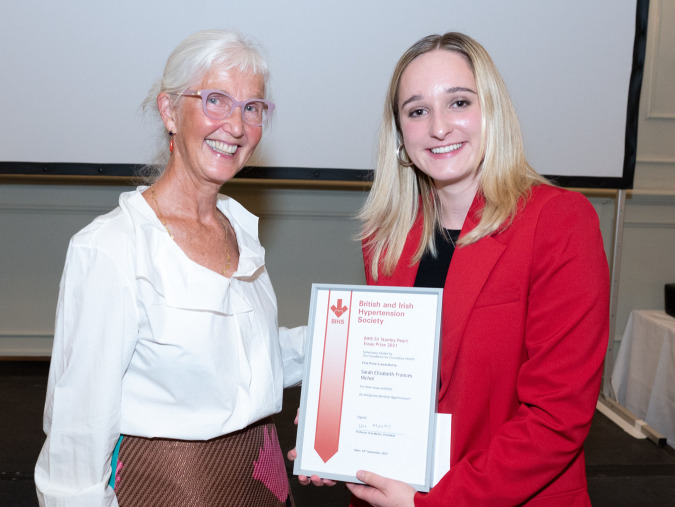
Photograph at the awards presentation. Sir Stanley Peart’s daughter Celia Pett presenting the essay prize to Sarah Nichol at the BIHS Annual Scientific Meeting 2021 in Brighton.

This essay won the first place in the British and Irish Hypertension Society Sir Stanley Peart Essay competition (Fig. 2).

*In order of appearance:*

*Sarah Nichol*

*George Pickering*

*David Hume*

*Ren*é *Descartes*

*Karl Popper*

*Immanuel Kant*

*Plato*

*Blaise Pascal*
